# MicroRNA-449a Enhances Radiosensitivity in CL1-0 Lung Adenocarcinoma Cells

**DOI:** 10.1371/journal.pone.0062383

**Published:** 2013-04-17

**Authors:** Yi-Jyun Liu, Yu-Fen Lin, Yi-Fan Chen, En-Ching Luo, Yuh-Ping Sher, Mong-Hsun Tsai, Eric Y. Chuang, Liang-Chuan Lai

**Affiliations:** 1 Graduate Institute of Physiology, National Taiwan University, Taipei, Taiwan; 2 Graduate Institute of Biomedical Electronics and Bioinformatics, National Taiwan University, Taipei, Taiwan; 3 Graduate Institute of Clinical Medical Science, China Medical University, Taichung, Taiwan; 4 Institute of Biotechnology, National Taiwan University, Taipei, Taiwan; 5 Bioinformatics and Biostatistics Core, Center of Genomic Medicine, National Taiwan University, Taipei, Taiwan; 6 YongLin Biomedical Engineering Center, National Taiwan University, Taipei, Taiwan; Boston University Medical Center, United States of America

## Abstract

Lung cancer is the leading cause of cancer-related mortality worldwide. Radiotherapy is often applied for treating lung cancer, but it often fails because of the relative non-susceptibility of lung cancer cells to radiation. MicroRNAs (miRNAs) have been reported to modulate the radiosensitivity of lung cancer cells and have the potential to improve the efficacy of radiotherapy. The purpose of this study was to identify a miRNA that can adjust radiosensitivity in lung adenocarcinoma cells. Two lung adenocarcinoma cell lines (CL1-0 and CL1-5) with different metastatic ability and radiosensitivity were used. In order to understand the regulatory mechanisms of differential radiosensitivity in these isogenic tumor cells, both CL1-0 and CL1-5 were treated with 10 Gy radiation, and were harvested respectively at 0, 1, 4, and 24 h after radiation exposure. The changes in expression of miRNA upon irradiation were examined using Illumina Human microRNA BeadChips. Twenty-six miRNAs were identified as having differential expression post-irradiation in CL1-0 or CL1-5 cells. Among these miRNAs, miR-449a, which was down-regulated in CL1-0 cells at 24 h after irradiation, was chosen for further investigation. Overexpression of miR-449a in CL1-0 cells effectively increased irradiation-induced DNA damage and apoptosis, altered the cell cycle distribution and eventually led to sensitization of CL1-0 to irradiation.

## Introduction

Lung cancer ranks first among cancer-related causes of death during the past few decades in Taiwan, and the mortality of lung cancer is increasing annually. Lung cancer can be classified into two major groups: small cell lung cancer (SCLC) and non-small cell lung cancer (NSCLC). The latter group is further divided into subtypes of squamous cell carcinoma, large cell carcinoma and adenocarcinoma. Among these three, adenocarcinoma is the most common subtype and has a high mortality rate. The survival rate at 5 years is generally less than 15% [1]. For patients with locally advanced NSCLC, radiotherapy is usually regarded as the treatment of choice. However, cellular response to irradiation is complex. Also, the treatment effects depend on many factors. For example, the dose, dose rate, and fractionation play an equally important role in deciding the fate of the cell. One of the main causes of failure in radiotherapy is radioresistance [2]. Therefore, a better understanding of how radioresistance is developed at the molecular level is needed to develop effective radiotherapy strategies in the future.

MicroRNAs (miRNAs) are small endogenous non-coding RNAs that play crucial regulatory roles in gene expression by targeting mRNAs for translation inhibition and/or degradation of mRNA. Mature miRNAs, containing ∼22 nucleotides, originate from longer primary miRNA transcripts, and are processed into mature form through two steps of endonuclease cleavage. The miRNA-induced silencing complex (miRISC) mediates miRNA-induced regulation of mRNA by docking at the 3′-untranslated region (3′-UTR) of a target gene complementary to the seed sequence of the miRNA, resulting in target mRNAs cleavage or translation inhibition [3]. It has been estimated that miRNAs regulate approximately 30% of human genome that contains potential miRNA binding sites in their 3′-UTR, and one miRNA can target multiple mRNAs [4]–[6]. Thus, miRNA serves as a regulator which simultaneously modulates different pathways by targeting different mRNAs. MiRNAs have been implicated in diverse cellular and developmental processes, and several recent studies showed that miRNA expression is often dysregulated in cancer, where mirRNAs can function as tumor suppressors or oncogenes [7], [8]. In addition, it has been reported that miRNA expression is affected by irradiation [9]–[12]. More and more evidence has confirmed that miRNAs can modulate the radiosensitivity of cancer cells, suggesting the potential to improve the efficacy of radiotherapy [13]–[18].

To better understand the mechanisms underlying invasiveness and metastasis, five lung adenocarcinoma sublines (CL1-1, CL1-2, CL1-3, CL1-4 and CL1-5) displaying progressive invasiveness and metastatic capabilities were obtained through the in vitro selection process [19]. Among these cell lines, CL1-5 is the most aggressive, and has been preferentially used for comparison to CL1-0 in studies of cancer progression and metastasis [20]–[23]. However, the radiation response of CL1-0 and CL1-5 has not been explored. Here, we found that CL1-0 and CL1-5 have different radiosensitivity, with more radioresistance in CL1-0. Hence, the purpose of this study was to use these two lung adenocarcinoma cell lines to identify the miRNAs regulating radiosensitivity and to examine the effect of miRNAs on radioresponse.

Based on the results of miRNA microarrays and literature surveys, we focused on miR-449a. MiR-449a, sharing the same seed sequence with tumor suppressors miR-34 family [24], was reported to provoke cell cycle arrest [25], [26] as well as induce apoptosis in prostate and gastric cancers [25], [27], [28]. Moreover, miR-449a was found to be strongly expressed in lung tissue [29], but lower amounts in lung cancer tissues [30]. Reintroduction of miR-449 in tumor cells efficiently drives them into cell cycle arrest and apoptosis [25], [29], [31]. Therefore, we further demonstrated that, after irradiation exposure, overexpression of miR-449a further enhanced irradiation-induced DNA damage and apoptosis, altered the cell cycle distribution, and consequently sensitized the radioresistant CL1-0 cells to irradiation.

## Materials and Methods

### Cell culture, plasmid and microRNA transfection

The lung adenocarcinoma cell lines, CL1-0 and CL1-5, were established by Chu et al. and were gifts from Dr. Pan-Chyr Yang (National Taiwan University, Taipei, Taiwan) [19]. CL1-5 was a more aggressive cell line selected by transwell assay from its parent strain, and CL1-0 was less invasive. Cell lines were grown in RPMI 1640 media (Invitrogen, Carlsbad, CA, USA) supplemented with 10% fetal bovine serum (GIBCO, Carlsbad, CA, USA) and 1% antibiotics (GIBCO), and maintained in a humidified atmosphere of 5% CO_2_ and 95% air at 37°C.

To generate a construct expressing miR-449a, miRNA expressing plasmids were created using the BLOCK-iT pol II miR RNAi Expression Vector Kit with EmGFP (Invitrogen). The primary miRNA sequence of miR-449a with flanking regions was obtained by PCR, and was inserted into the Block-iT Pol II miR RNAi Expression Vector, pcDNA6.2-GW/EmGFP-miR. For transfection, CL1-0 cells were seeded in antibiotic-free medium at a density of 30–40%. Transfection was performed with *Trans*IT®-2020 Transfection Reagent (Mirus Bio LLC, Madison, WI, USA) according to manufacturer's protocol.

### RNA extraction, reverse transcription, and real-time PCR quantification

Both CL1-0 and CL1-5 were treated with 10 Gy of irradiation, and were harvested after radiation exposure at different time points as indicated. Total RNA of irradiated CL1-0 and CL1-5 cells was extracted using TRIZOL reagent (Invitrogen) according to the manufacturer's protocol. MiRNA was reverse transcribed into cDNA using the TaqMan MicroRNA Reverse Transcription kit (Invitrogen), and the primer used for miR-449a was 5′GTCGTATCCAGTGCAGGGTCCGAGGTATTCGCACTGGATACGATACCAGCT3′. Real-Time PCR was performed using a 7900 Fast Real-Time PCR system (Applied Biosystems, Carlsbad, CA, USA) with miR-449a-specific primers (Forward: 5′TGGCGGTGGCAGTGTATTGTTA3′; Reverse: 5′GTGCAGGGTCCGAGGT3′), Universal ProbeLibrary Probe #21 (Roche, Germany), and KAPA PROBE FAST qPCR Master Mix (KAPA BIOSYSTEMS, Boston, MA, USA).

Total RNA was transcribed into cDNA with random primers using the High Capacity cDNA Reverse Transcription kit (Invitrogen) for *HDAC1* mRNA detection. Primers used for *HDAC1* were 5′ATCTGCTCCTCTGACAAACGA3′ (forward) and 5′CGGTGACTTCTTTCTTCTCCT3′ (reverse). The resulting cDNA was detected using Power SYBR® Green PCR Master Mix (Applied Biosystems) with 7900 Fast Real-Time PCR system (Applied Biosystems). *RNU44* and *GAPDH* were used as endogenous controls to normalize miRNA and mRNA, respectively. Primers used for *RNU44* were 5′TCGCGCCTGGATGATGATAGC3′ (forward) and 5′GTGCAGGGTCCG AGGT3′ (reverse). Primers used for *GAPDH* were 5′TGCACCACCAACTGCTTAG3′ (forward) and 5′GATGCAGGGATGATGTTC3′ (reverse).

### Microarray data analysis

Illumina Human microRNA BeadChips (Illumina, San Diego, CA, USA) were used to profile miRNA expression after irradiation. Quantile normalization was performed using Partek Genomics Suite software (Partek, St. Louis, MO, USA). Fold change (>1.5×) and t-tests (P<0.05) of each time point as compared to time 0 were applied for selecting radioresponsive miRNAs. Microarray data of this study are MIAME compliant, and have been submitted to the MIAME compliant GEO (Gene Expression Omnibus) database (accession number GSE40602).

### Flow cytometry analysis of cell cycle and apoptosis

Cells plated at 50–60% confluency were irradiated with 10 Gy and grown for 0–48 h. Samples were trypsinized, resuspended in phosphate-buffered saline (PBS) (GIBCO, Carlsbad, CA, USA), and fixed with cold 70% ethanol overnight. The DNA contents were evaluated after staining with propidium iodide (PI) solution containing 50 µg/ml PI (Sigma, St. Louis, Mo, USA), 0.1 mg/ml RNase A (Sigma), 0.05% Tritin X-100 (Sigma) in PBS (GIBCO). Cell cycle analysis was carried out using a FACScan flow cytometer (Becton Dickinson, San Diego, CA, USA) and CellQuest software.

The FITC Annexin V Apoptosis Detection Kit (BD Pharmingen, San Jose, CA, USA) was used to detect apoptosis cells by flow cytometry. Cells were exposed to 10 Gy of irradiation, and were harvested at different time points for 48 h after exposure. Annexin V binding buffer was used to resuspend cells, and the cell suspensions were stained with FITC-annexin V and PI staining solution for 15 min at room temperature. The apoptotic/necrotic cell population was analyzed with a FACSCalibur flow cytometer (Becton Dickinson). The irradiation-induced apoptosis was calculated by subtracting the percentage of annexin V-positive cells with irradiation from that of the corresponding group without irradiation.

### Clonogenic assay

Cells were plated at 50–60% confluency overnight, and were irradiated with 0 to 10 Gy as indicated. Due to different radiosensitivity, 500 CL1-0 cells and 10,000 CL1-5 cells were seeded into 6 cm dishes. Cells were incubated for 8 days and then washed with PBS, fixed with 100% ethanol and stained for 1 min with 0.1% crystal violet. Colonies with ≧50 cells were counted manually. The surviving fraction was calculated using the following equation: surviving fraction  =  colonies counted/(cells plated × plating efficiency). Plating efficiency was defined as: (mean colony counts/cells seeded for unirradiated controls) × 100%.

### Western blot

Cells were washed twice with PBS (GIBCO), and lysed in RIPA lysis buffer (Sigma). Protein concentrations were determined by a standard protein assay (Bio Rad Laboratories, Hercules, CA, USA). Protein (70 µg) samples were loaded on 15% SDS gels. After electrophoresis, proteins were transferred to nitrocellulose membranes. Blocking was performed with 3% nonfat milk in Tris-buffered saline. Membranes were incubated overnight with the following antibodies: γH2AX (Millipore, Billerica, MA, USA), CASP3 (Cell Signaling Technology, Danvers, MA, USA), HDAC1 (Millipore), and ACTB (GeneTex, Irvine, CA, USA). After washing and incubating with secondary antibodies, blotted protein detection was performed by enhanced chemiluminescence (Millipore) with the BioSpectrum Imaging System (UVP, Upland, CA, USA).

### MTT assay

Cells were seeded at a density of 2.5 × 10^4^ cells/250 µl in a 24-well plate. After overnight incubation, cells were irradiated and allowed to grow for 48 h. After washing twice with PBS, cells were incubated with MTT (3-(4,5-dimethylthiazol-2-yl)-2,5-diphenyltetrazolium bromide) solution (Sigma) (1 ml/well of 5 mg/ml solution in PBS) for 4 h. Next, 200 µl/well DMSO (Sigma) was added to dissolve the converted purple formazan, and the absorbance of formazan was measured at 570 nm using an ELASA reader (BioTek, Winooski, VT, USA).

## Results

### CL1-0 cells are more radioresistant than CL1-5

**Figure 1:**
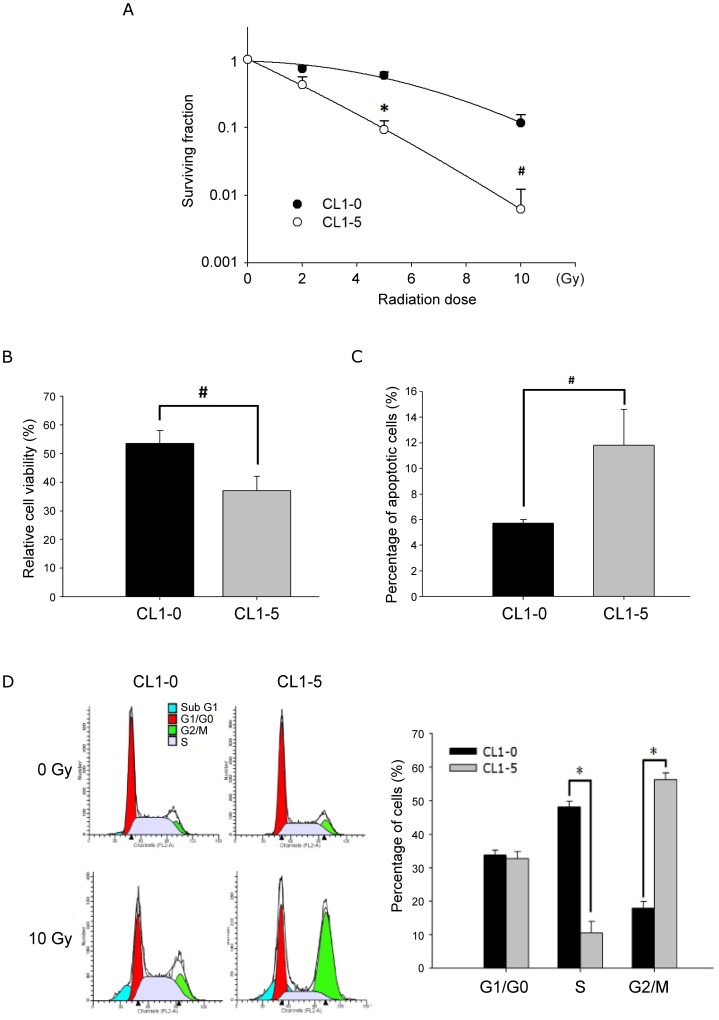
Differential irradiation responses in CL1-0 and CL1-5. (A) Clonogenic assays of CL1-0 and CL1-5 cells treated with 0, 2, 5, and 10 Gy radiation. The surviving fraction was measured 8 days post-irradiation. (B) MTT assays for cells at 48 h after 10 Gy radiation. Cell viability is expressed as percentage relative to un-irradiated control cells. (C) Flow cytometry analysis of apoptosis using annexin V and propidium iodide in cells treated with 10 Gy. The bar chart shows the percentage of apoptotic cells induced by irradiation. (D) Flow cytometry analysis of cell cycle using propidium iodide at 24 h post-irradiation. The proportions of cells in each phase of the cell cycle were quantitated using ModFit LT software. Left: one representative diagram, with different colors indicating different phases. Right: quantitative bar chart. Data are represented as mean ± SD for three independent experiments. #: *P*<0.05, *: *P*<0.001.

CL1-0 and CL1-5 were regarded as a good model for metastasis-associated studies, because CL1-5 cells are more aggressive than non-metastatic CL1-0 cells. But the radioresponses of CL1-0 and CL1-5 have not been investigated. Therefore, clonogenic assays were performed on these two cell lines after administration of 0, 2, 5, 10 Gy. Intriguingly, clonogenic survival revealed that CL1-0 cells were more resistant to irradiation than CL1-5 cells (*P*<0.001; Fig. 1A). MTT assays also demonstrated that cell viability of CL1-0 was higher than that of CL1-5 upon irradiation (Fig. 1B), which suggests that CL1-0 is more radioresistant than CL1-5.

In order to characterize the mechanism involved in the different radiosensitivities of CL1-0 and CL1-5, flow cytometry was used to examine apoptosis and cell cycle distribution post-irradiation. An annexin V binding assay was used to evaluate irradiation-induced apoptosis, which was calculated by subtracting the percentage of annexin V-positive cells with irradiation from that of the corresponding group without irradiation. As shown in Fig. 1C, the CL1-5 had significantly (*P*<0.05) more apoptotic cells than the CL1-0 post-irradiation. Moreover, as shown in the left panel of Fig. 1D, cell cycle analysis indicated that cell cycle distribution pattern of CL1-5 was different from that of CL1-0 at 24 h after irradiation. In CL1-5 cells, the proportion of cells in the G2/M phase was dramatically increased, and the proportion of cells in the S phase was decreased as compared with CL1-0 (Fig. 1D). The results of flow cytometry indicated that different radiosensitivities between CL1-0 and CL1-5 resulted from differential regulation of apoptosis and cell cycle progression.

### Identification of microRNA regulating radiosensitivity

**Figure 2:**
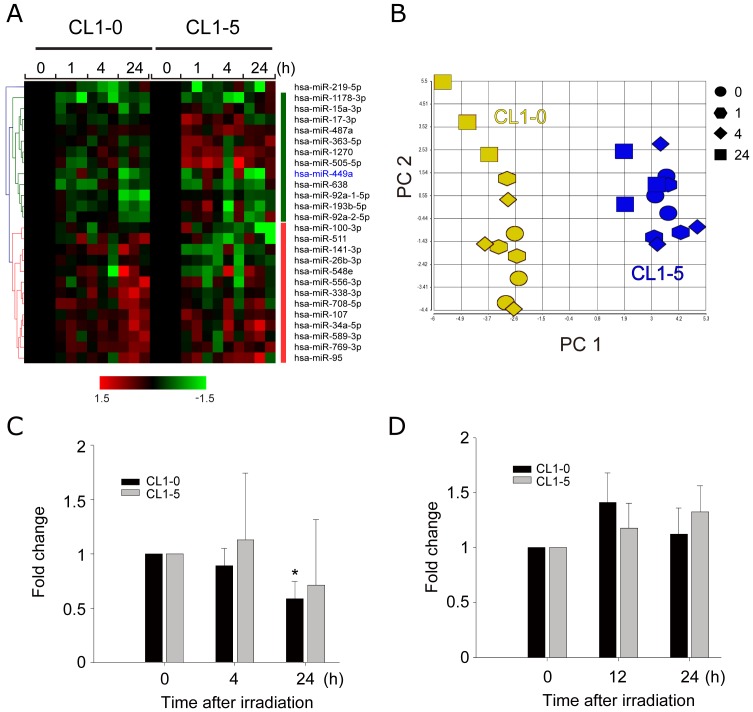
Dynamics of differentially expressed miRNA after irradiation in CL1-0 and CL1-5. (A) Hierarchical clustering of differentially expressed miRNA from CL1-0 and CL1-5 treated with 10 Gy. Cells were harvested respectively at 0, 1, 4, 24 h after irradiation. (B) Principal component analysis (PCA) of the radiation-responsive genes. The axes are the first two principal components (PC), which can explain most of the miRNA expression profiling. Three independent experiments were done at each time point. Different colors represent different cells; different shapes represent different time points. (C) Relative miR-449a expression level measured by microarray. Data were the mean ± SD for three independent experiments. *: *P*<0.05. (D) Relative miR-449a expression level measured by quantitative RT-PCR. Data were the mean ± SD for four independent experiments.

In order to identify miRNAs whose expression was dysregulated after irradiation and which could alter the radiosensitivity of cancer cells, we measured the miRNA expression profile in CL1-0 and CL1-5 post-irradiation. CL1-0 and CL1-5 were treated with 10 Gy and harvested after 0, 1, 4, and 24 h. Each time series was done in triplicate. The genomic profiling of miRNAs was measured using Illumina Human microRNA Beadchips. Quantile normalization was performed to adjust background signals. To identify differentially expressed miRNAs, Student's t-test was used to examine the expression levels of every time point after irradiation compared to that of time zero. The intensities of miRNAs lower than the 10^th^ intensity percentile were regarded as noise, and the corresponding miRNAs were excluded from further analysis. The criteria for selecting radiosensitivity-related miRNAs were fold change >1.5× and a significant difference (*P*<0.05) between a given time point and time zero. We identified 26 registered miRNAs in miRBase [32] from 1,145 miRNA probes in the Illumina microarray platform. Among them, 52% (n = 15) were up-regulated and 48% (n = 11) were down-regulated post-irradiation (Fig. 2A). All of them were identified at only one time point, and most (52%) were identified at the 24 h time point.

Principal component analysis (PCA) was performed to examine whether the differentially expressed miRNAs could really distinguish irradiated from un-treated samples. As shown in Fig. 2B, dots with different shapes indicate different time points, and different color indicates different cell lines. Samples of different time points in CL1-5 (blue spots in Fig. 2B) are clustered together, indicating the expression profiles of these miRNAs did not change after radiation. In contrast, the expression profiles of miRNAs at 24 h after radiation in CL1-0 (yellow squares in Fig. 2B) were different from non-irradiation control (yellow circles). That is, the expression patterns in CL1-0 at the time points 0, 1, 4 h post-irradiation were similar, but were different from that at 24 h post-irradiation, indicating that these identified miRNAs responding to irradiation were activated at 24 h. Since the radio-responsiveness of these differentially expressed miRNAs was only observed in CL1-0, not CL1-5, miRNAs from CL1-0 were chosen for further experiments.

Since cellular response to radiation is related to apoptosis and cell cycle arrest, we investigated whether the target genes of these differentially expressed miRNAs were involved in apoptosis and cell cycle arrest using an online bioinformatics tool miRSystem [33]. As shown in Table 1, we identified 11 miRNAs whose predicted target genes were involved in apoptosis or cell cycle, and most of these miRNA had references to support the prediction (data not shown). Among these miRNAs, we focused on miR-449a for further experiments to investigate whether miR-449a could regulate radiosensitivity based on following reasons. First, the results of both microarray and quantitative RT-PCR showed that expression levels of miR-449a in CL1-5 cells did not respond to irradiation (Fig. 2C & D). Although miR-449a in CL1-0 cells did not significantly down-regulated at 24 h post irradiation using qRT-PCR, we observed significant (*P*<0.05) down-regulation of miR-449a in CL1-0 cells at 24 h post-irradiation (Fig. 2C). Also, it has been reported that miR-449a plays important roles in regulating cell cycle progression, cell proliferation, and apoptosis in many types of cancer [25].

**Table 1:** Predicted targets of significant miRNAs involved in cell cycle or apoptosis.

			Functional Categories			
miRNA*	Total Predicted Targets	Database^§^	Term	Total Genes	Predicted Targets	*P*-value^#^
miR-95	27	KEGG	Cell cycle	124	2	9.62E-03
miR-107	783	KEGG	Cell cycle	124	13	3.45E-04
		Pathway interaction database	Polo-like kinase signaling events in the cell cycle	106	9	8.82E-03
		Reactome	Cell cycle mitotic	330	19	1.28E-02
		Reactome	Apoptosis	148	10	2.21E-02
miR-141-3p	837	KEGG	Cell cycle	124	13	6.23E-04
		Reactome	Cell cycle mitotic	330	19	2.07E-02
miR-219-5p	298	Pathway interaction database	Caspase cascade in apoptosis	46	3	2.09E-02
		Reactome	Apoptosis	148	5	3.55E-02
		Pathway interaction database	Polo-like kinase signaling events in the cell cycle	106	4	4.14E-02
miR-338-3p	364	Reactome	Intrinsic pathway for apoptosis	30	3	1.17E-02
miR-34a-5p	716	KEGG	Cell cycle	124	11	1.69E-03
		Reactome	Cell cycle mitotic	330	19	6.14E-03
		Reactome	Apoptosis	148	9	2.99E-02
		Pathway interaction database	Caspase cascade in apoptosis	46	4	4.55E-02
miR-449a	659	KEGG	Cell cycle	124	9	8.19E-03
		Reactome	Intrinsic pathway for apoptosis	30	4	9.95E-03
		Pathway interaction database	Caspase cascade in apoptosis	46	4	3.65E-02
		Reactome	Cell cycle mitotic	330	14	4.92E-02
miR-487a	199	Reactome	Cell cycle mitotic	330	6	4.89E-02
miR-511	612	KEGG	Cell cycle	124	9	5.40E-03
		Reactome	Regulation of mitotic cell cycle	82	7	5.82E-03
		Reactome	Apoptosis	148	9	1.43E-02
miR-556-3p	148	Reactome	Cell cycle mitotic	330	6	1.73E-02
miR-769-3p	68	Reactome	Apc c-mediated degradation of cell cycle proteins	82	2	2.46E-02
		Pathway interaction database	Polo-like kinase signaling events in the cell cycle	106	2	3.83E-02
		KEGG	Cell cycle	124	2	4.97E-02

* miRNAs are listed in numeral order. ^§^ KEGG: http://www.genome.jp/kegg/; Pathway interaction database: http://pid.nci.nih.gov/; Reactome: http://www.reactome.org/ReactomeGWT/entrypoint.html. ^#^ P-value <0.05.

### MiR-449a sensitizes CL1-0 cells to DNA damage post-irradiation

**Figure 3:**
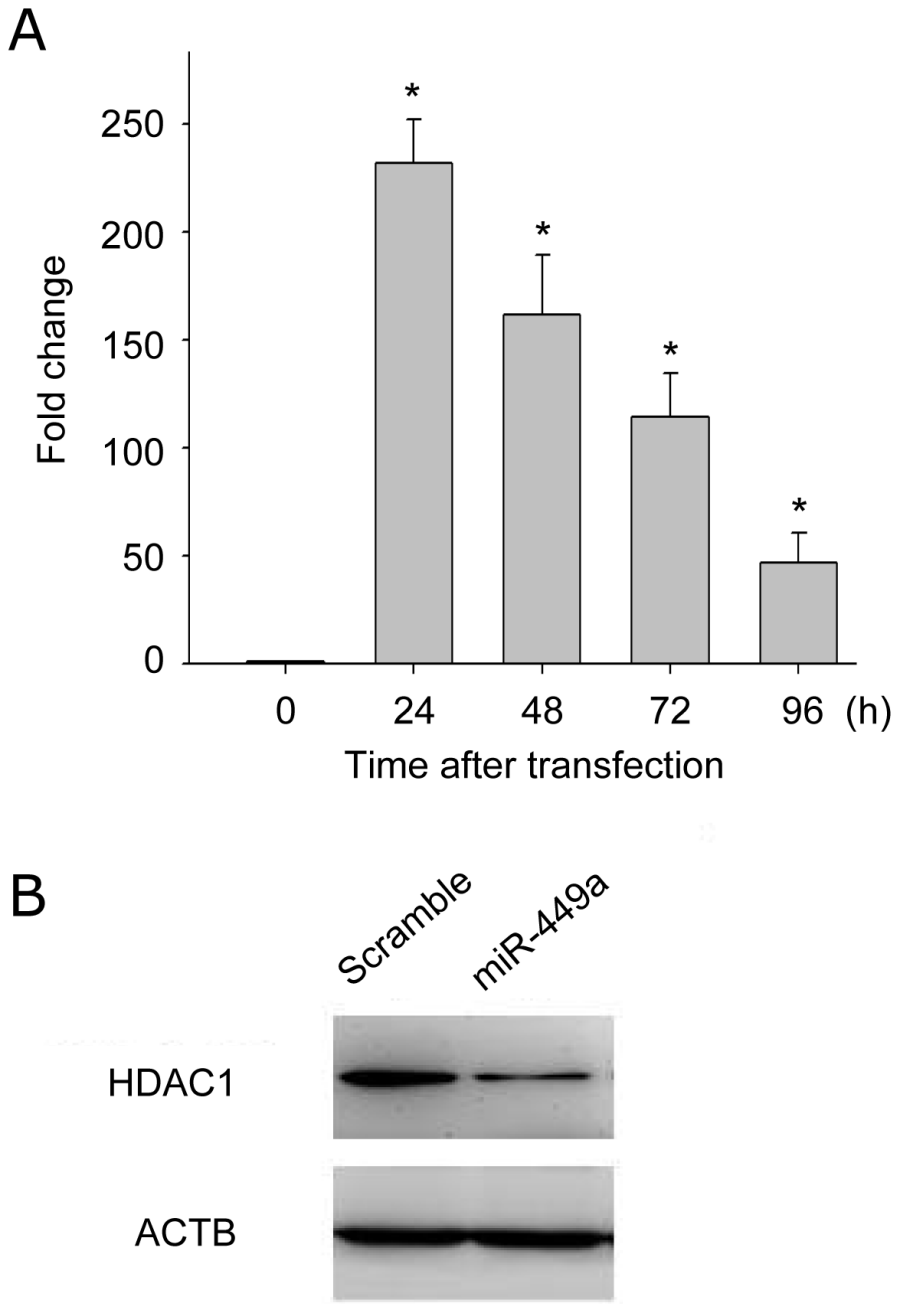
Successful over-expression of miR-449a in CL1-0. (A) Expression profiling of miR-449a over-expressed in CL1-0. Relative expression levels of miR-449a in CL1-0 cells were measured at indicated time points by real-time PCR after transiently transfected with miR-449a expression plasmid. *RNU44* served as an endogenous control. Each measurement was made in triplicate. *: *P*<0.001. (B) Immunoblotting of histone deacetylase 1 (HDAC1), a validated target of miR-449a. Protein levels of HDAC1 in CL1-0 were analyzed 72 h after transfection. ACTB (β-actin) was a loading control.

In order to investigate the role of miR-449a in modulating the radiosensitivity of CL1-0, miR-449a was overexpressed in CL1-0 cells using a miRNA expression plasmid containing a primary miR-449a sequence. After transfection, the relative expression level of miR-449a was significantly increased, as measured by real-time PCR (Fig. 3A). At 96 h after transfection, there was still ∼50-fold induction. Furthermore, a western blot confirmed that the protein level of one of miR-449a's targets, histone deacetylase 1 (HDAC1) [27], was strongly down-regulated upon miR-449a overexpression (Fig. 3B).

**Figure 4:**
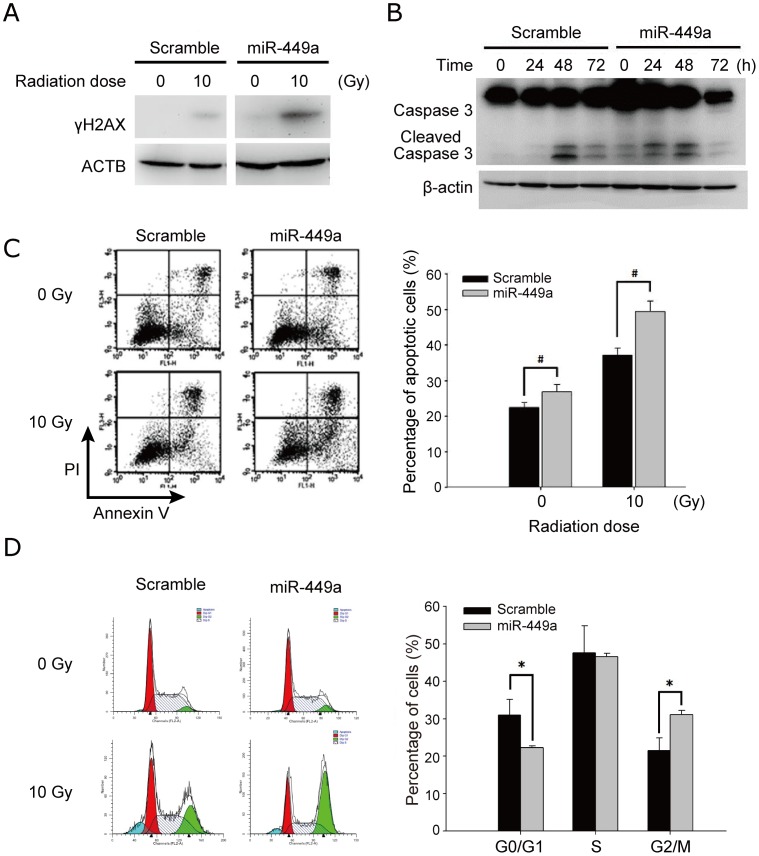
Over-expression of miR-449a in CL1-0 increased radiosensitivity. (A) Immunoblotting of γH2AX in miR-449a-overexpressing cells. Cells were harvested 1 h after irradiation. ACTB (β-actin) was a loading control. (B) Immunoblotting of caspase 3 in miR-449a-overexpressing cells. Cells were harvested at 0, 24, 48, and 72 h after irradiation. β-actin was a loading control. (C) Flow cytometry analysis for apoptosis using annexin V and propidium iodide in miR-449a-overexpressing cells treated with 10 Gy. At 48 h post-irradiation, miR-449a-overexpressing cells were stained with annexin V and propidium iodide, and the percentage of apoptotic cells was calculated. One representative diagram is shown to the left, and the percentage of annexin V- positive cells after irradiation is shown to the right. Data in the bar chart are the mean ± SD for three independent experiments. (D) Flow cytometry analysis for cell cycle using propidium iodide in miR-449a-overexpressing cells after irradiation. Flow cytometry analysis of cell cycle progression was performed in miR-449a-overexpressing cells 24 h after treated with 10 Gy. The proportions of cells in each phase of cell cycle were quantitated using ModFit LT software. Left: one representative diagram, with different colors indicating different phases. Right: quantitative bar chart. Data in the bar chart are the mean ± SD for three independent experiments. #: *P*<0.05,*: *P*<0.001.

It is known that irradiation causes DNA double-strand breaks (DSBs), and DSBs induce phosphorylation of H2AX at serine 139 (γH2AX). We used immunoblotting of γH2AX to examine the effect of miR-449a on irradiation-induced DNA damage. Forty-eight hours post-transfection, cells were irradiated with 10 Gy and collected after 1 h for western blot analysis. After irradiation, a significant increase in the level of γH2AX was observed in CL1-0 cells overexpressing miR-449a (Fig. 4A). These results suggest that miR-449a is involved in sensitization of CL1-0 to irradiation via augmenting irradiation-induced DSBs.

### Irradiation-induced apoptosis is enhanced by miR-449a

After irradiation, if DNA damage is not repaired properly, cells may proceed with apoptosis. Since miR-449a sensitized CL1-0 to irradiation, and augmented irradiation-induced DSBs, we further investigated the effect of miR-449a on cell death post-irradiation. First, activation of caspase 3, the key step of apoptosis, was examined. As expected, over-expression of miR-449a alone increased the cleaved form of caspase 3. Furthermore, activation of caspase 3 by cleavage occurred earlier in miR-449a-overexpressing CL1-0 cells treated with irradiation (24 h) than in control cells (48 h) (Fig. 4B). The irradiation-evoked caspase 3 activation continued to increase up to 48 h and attenuated at 72 h in miR-449a-expressing cells.

Next, miR-449a-overexpressing CL1-0 cells were treated with 10 Gy and subjected to annexin V binding assay at 48 h post-irradiation. As shown in Fig. 4C, one representative diagram is shown to the left, and the percentage of annexin V- positive cells after irradiation is shown to the right. Without radiation, cells overexpressing miR-449a exhibited more apoptosis as compared to control cells. Moreover, when cells exposed to radiation, although more apoptotic cells were found both in miR-449a-overexpressing and control cells, the amount of apoptosis induced by irradiation in miR-449a-overexpressing cells was significantly greater than that in control cells (Fig. 4C). These results suggested that miR-449a sensitizes CL1-0 cells to irradiation by enhancing irradiation-induced apoptosis.

### MiR-449a enhances G2/M arrest post-irradiation

MiR-449a was shown to provoke cell cycle arrest [24], [25], [34]. Furthermore, after irradiation, DNA damage transiently halts the cell cycle for repair. The alteration of cell cycle progression post-irradiation correlated closely with radiosensitivity, which prompted us to further examine the effect of miR-449a on cell cycle progression post-irradiation. As shown in Fig. 4D, at 24 h after treatment with 10 Gy, one representative diagram is shown to the left. The proportions of cells in each phase of cell cycle were indicated by different colors and quantitated using ModFit LT software. Ectopic expression of miR-449a increased the proportion of CL1-0 cells in the G2/M phase and decreased the proportion of cells in G0/G1 phase compared with that of control cells (Fig. 4D). These results implied that miR-449a caused CL1-0 cells to accumulate in G2/M phase post-irradiation.

### Over-expression of miR-449a efficiently suppresses cell viability post-irradiation

**Figure 5:**
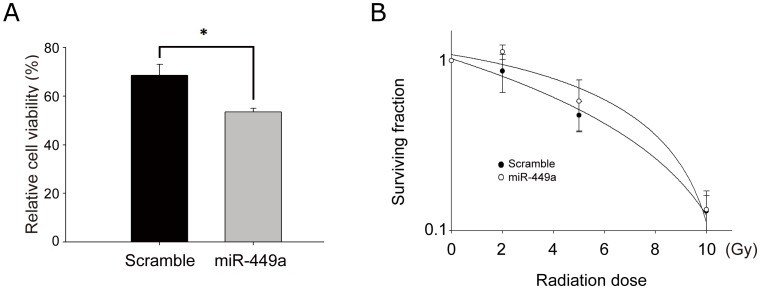
miR-449a reduced cell viability post-irradiation in MTT assays. (A) MTT assays for miR-449a-overexpressing cells were performed at 48 h post-irradiation with 10 Gy. Cell viability is expressed as percentage relative to un-irradiated controls. Data are the mean ± SD for four independent experiments. *: *P*<0.001. (B) Clonogenic assays of miR-449a-overexpressing CL1-0 cells treated with 0, 2, 5, and 10 Gy radiation. The surviving fraction was measured 8 days post-irradiation.

Since it has been demonstrated that miR-449a induced more severe DNA damage and more apoptosis after irradiation, we hypothesized that the survival capability of cells post-irradiation might be altered upon miR-449a transfection. Hence, MTT and clonogenic assays were performed to examine the effect of miR-449a on post-irradiation cell survival. In Fig. 4B&C, we have shown that, without irradiation, overexpression of miR-449a alone caused increase in apoptosis. In addition, we observed that, following irradiation in the presence of ectopic miR-449a, cell viability was further suppressed at 48 h as compared with un-irradiated cells in the MTT assay (Fig. 5A), while there was no significant difference in clonogenic survival (Fig. 5B).

## Discussion

In this study, we demonstrated that two lung adenocarcinoma cell lines with an isogenic background, CL1-0 and CL1-5, displayed different radioresponses, and identified miR-449a as a regulator of their radiosensitivity. MiR-449a sensitized radioresistant CL1-0 cells to irradiation through augmentation of DNA damage, enhancement of apoptosis, and increase of G2/M phase arrest post-irradiation.

Previous studies showed that CL1-0 and CL1-5, although derived from the same parental cell line, displayed differences in invasiveness and metastatic potential [19]. Although many molecules, such as caveolin-1, CRMP-1 and CTGF, have been reported as being important in promoting or inhibiting invasion and metastasis of lung cancer based on the cell line series [35]–[37], this model has not been used in radiation-related studies to date. In the current study, these two cell lines were used to explore the mechanisms of radiosensitivity for the first time. Functional assays showed that CL1-0 and CL1-5 response to irradiation treatment with distinguishing ways which contributed to different cell fates post irradiation (Fig. 1). CL1-5 post irradiation is more radio-sensitive with lower survival rate (Fig. 1A and 1B), more radiation-induced apoptosis (Fig. 1C) and altered cell cycle progression (Fig. 1D) than CL1-0.

Conventionally, it was believed that patients who experienced local failure following radiation treatment had higher rate of distant metastasis [38]. That is, the progression of tumors to increased levels of malignancy was related to the increased ability to become radioresistant. However, Suit *et al*. reported that there was no correlation between the radiation sensitivity of clinical or xenografted tumors and metastatic activity [39]. Furthermore, we showed that more metastatic and aggressive lung cancer cells have higher radiosensitivity. To investigate whether this phenomenon was limited to this specific lung cancer cell line, we examined the radiosensitivity in a more aggressive breast cancer cell line MDA-MB-231 and a less metastatic one MCF-7. Similarly, highly metastatic MDA-MB-231 cells having more radiosensitive than poorly metastatic MCF-7 cells (Fig. S1). This phenomenon was also reported in other cancers, such as breast cancer [40] and melanoma [41], which are consistent with our findings. Yet, more experiments are still needed to investigate its mechanism.

Previous studies have suggested that radiation causes altered miRNA expression in several tumor lines, such as glioblastoma [42], lung cancer [9], and prostate cancer [43]. Our results not only confirmed that radiation noticeably induced changes in the expression of miRNAs, but also extended the findings that the activation period was at 24 h post-irradiation in lung cancer cell lines. These results indicated that altered miRNAs after radiation are involved in the mechanism of radiation response.

Several studies have reported that miRNAs can modulate the radiosensitivities via the following mechanisms. For example, miR-101 could sensitize human tumor cells to radiation by targeting ATM and DNA-PK catalytic subunit (DNA-PKcs) to inhibit DNA repair [44]. The overexpression of miR-34b increased radiosensitivity by significantly reducing BCL2 expression and cell survival, but no significant difference in apoptotic cell population or the cycle profile [45]. Over-expressing miRNA-7 could radiosensitize A549 lung cancer cells by activating EGFR-associated signaling [16]. MiRNA-148b enhanced the radiosensitivity of non-Hodgkin's Lymphoma cells by promoting radiation-induced apoptosis [46]. In order to further investigate the mechanism of radiation response, one of the miRNA candidates screened from microarrays was selected for further biological experiments.

Within the miRNA candidates identified at 24 h in CL1-0, miR-449a drew our attention. It has been reported that miR-449a is a direct target of E2F1 and prevents tumor cells from entering S phase by down-regulating E2Fs directly and by inhibiting the activity of E2F transcription factors through the reduction of CDKs [25], [26]. Also, miR-449a induces apoptosis in prostate and gastric cancers through the activation of p53 by down-regulating the histone deacetylase HDAC1 [27] and SIRT1 [25], [28]. In cells depleted of p53, miR-449a also induces apoptosis, though the underlying mechanisms are not fully understood [25]. Moreover, miR-449a was found to be strongly expressed in lung tissue, especially in differentiated bronchial epithelium, without any sign of cell death [29]; while tumor cells contained far lower amounts of miR-449a [30]. Reintroduction of miR-449 in tumor cells efficiently drives them into cell cycle arrest and apoptosis [25], [29]. This selective proapoptotic activity is compatible with the idea that miR-449 may be involved in tumor-suppressive activities.

Since we showed that the difference in radiosensitivity between CL1-0 and CL1-5 was attributed to different mechanisms of apoptosis and cell cycle progression post-irradiation, we hypothesized that miR-449a, known for regulating apoptosis and cell cycle arrest, might be a regulator of radiosensitivity. Thus, we focused on miR-449a for further experiments.

Our results showed that cell cycle progression might be one of the factors modulating the radiosensitivity. A significant difference in the proportion of CL1-0 cells at G2/M phase was observed at 24 h post-irradiation, indicating that one of the mechanisms that activation period at 24 h post-irradiation may be due to the G2/M arrest. After miR-449a transfection following irradiation treatment, the altered cell cycle pattern of CL1-0 (Fig. 4D) was shifted to be similar to that of CL1-5 (Fig. 1D), which was identified as a radiosensitive cell line in this lung adenocarcinoma model and also displayed G2/M arrest pattern post irradiation. Radiation-induced G2/M arrest was previously observed 24 hours after irradiation in radiosensitive cell line [47]. One possible explanation of more dead cells after irradiation may be due to that cells are most radiosensitive in G2/M phases according to the law of Bergonie and Tribondeau. However, differences still existed between the cell cycle of miR-449a-overexpressed CL1-0 and that of CL1-5. For example, over-expression of miR-449a did not show any effect in S phase (Fig. 4D). In contrast, decreasing S population was observed in radiosensitive CL1-5 (Fig. 1D), which is consistent with previous reports in squamous cancer cells and lymphocytes [48], [49]. Furthermore, the decreasing G0/G1 population was observed in CL1-0 over-expressed with miR-449a, which was probably caused by the G2/M arrest (Fig. 4D).

Radiation-induced apoptosis is considered to be one of the main cell death mechanisms following exposure to irradiation [50]. MiRNAs have recently been shown to regulate apoptosis and may therefore contribute to radiation-induced cell death [51]. As expected, over-expression of miR-449a elevated the cleavage of caspase 3 without irradiation in CL1-0 cells (0 h in Fig. 4B). In addition, irradiation induced cleaved caspase 3 occurred earlier in miR-449a-overexpressing CL1-0 cells (Fig. 4B). Moreover, miR-449a sensitized the radio-resistant CL1-0 to irradiation, resulting in enhancement of irradiation-induced apoptosis by counting the proportion of Annexin V-positive cells (Fig. 4C). Previously, miR-449a was reported to induce apoptosis in prostate and gastric cancers through the activation of p53 by down-regulating the histone deacetylase HDAC1 [27]. Inhibition of HDAC1 was suggested to serve as a way of enhancing the radiosensitivity in lung cancer and esophageal cancer [52], [53]. Overall, we know the second mechanism of miR-449a modulating radiosensitivities is through regulation of apoptosis.

In addition to cell cycle and apoptosis, cellular response to radiation is related to DNA repair [54]. We observed that over-expression of miR-449a caused a subtle increase in the phosphorylation of H2AX even in the un-irradiated cells (Fig. 4A), suggesting DNA repair mechanism should increase. However, we did not observe any effect of miR-449a expression on radioresponse using unsynchronized clonogenic assays (Fig. 5B). We speculated that part of reasons was the effect of over-expressing miR-449a was transient, or those un-transfected cells might dilute the suppressive effect of miR-449a in clonogenic survival. Furthermore, since DNA damage activates checkpoint pathways that regulate specific DNA repair mechanisms in the different phases of the cell cycle [55], miR-449a might be only involved in DNA repair for a specific phase of cell cycle. Without synchronization, cells at different phases of the cell cycle can display different DNA repair mechanisms simultaneously during clonogenic assays. Therefore, future experiments should be done in synchronized cells.

However, in the current experiment setting, the transient effect of miR-449a overexpression deterred us from doing the synchronization of cell cycle. Furthermore, the pro-apoptotic activity of miR-449a in tumor cells makes it difficult to develop a stable clone to constitutively overexpress miR-449a. This impeded the establishment of a clear-cut mechanism of miR-449a in response to radiation. In the future, one of the alternatives is to establish an inducible system in stable clones, e.g. Tet-on/Tet-off. In such a system, miR-44a would be expressed only after stable clones are established. Hence, it could eliminate the problem of transfection efficiency, and will be a powerful tool for further examining the effect of miR-449a on radioresponse in lung adenocarcinoma cell lines.

In conclusions, the variable susceptibility of cells in response to radiotherapy is one of the main causes of radiotherapy failure. MiRNAs have been reported to modulate radiosensitivity and have the potential to improve the efficacy of radiotherapy. We used miRNA microarrays to screen the radiation-responding miRNAs in two isogenic lung adenocarcinoma cells with different radiosensitivity and found that miR-449a enhanced radiosensitivity by increasing irradiation-induced DNA damage and apoptosis and altering cell cycle distribution. This result is important for understanding how cancer cells acquire resistance to radiotherapy, and may lead to development of new therapeutic strategies for lung cancer. Also, further studies on the role of miR-449a in radiosensitivity in other resistant and radiosensitive cancer cell lines are another avenue to pursue.

## Supporting Information

Figure S1 **Differential irradiation responses in MCF7 and MDA-MB-231.** Clonogenic assays of MCF7 and MDA-MB-231 cells treated with 0, 0.5, 1, 2, and 4 Gy radiation. The surviving fraction was measured 8 days post-irradiation.
(TIF)

Table S1 **Normalized miRNA microarray data for CL1-0 and CL1-5.** Illumina Human microRNA BeadChips were used.
(XLS)

## References

[1] Jemal A, Siegel R, Ward E, Hao Y, Xu J, et al (2008) Cancer statistics, 2008. CA Cancer J Clin 58: 71–96. 18287387 10.3322/CA.2007.0010

[2] Rowell NP, O'Rourke NP (2004) Concurrent chemoradiotherapy in non-small cell lung cancer. Cochrane Database Syst Rev: CD002140. 10.1002/14651858.CD002140.pub2 15495029

[3] Bartel DP (2004) MicroRNAs: genomics, biogenesis, mechanism, and function. Cell 116: 281–297. 14744438 10.1016/s0092-8674(04)00045-5

[4] Ambros V (2004) The functions of animal microRNAs. Nature 431: 350–355. 15372042 10.1038/nature02871

[5] Lewis BP, Burge CB, Bartel DP (2005) Conserved seed pairing, often flanked by adenosines, indicates that thousands of human genes are microRNA targets. Cell 120: 15–20. 15652477 10.1016/j.cell.2004.12.035

[6] Friedman RC, Farh KK, Burge CB, Bartel DP (2009) Most mammalian mRNAs are conserved targets of microRNAs. Genome Res 19: 92–105. 18955434 10.1101/gr.082701.108 PMC2612969

[7] Esquela-Kerscher A, Slack FJ (2006) Oncomirs - microRNAs with a role in cancer. Nat Rev Cancer 6: 259–269. 16557279 10.1038/nrc1840

[8] Kloosterman WP, Plasterk RH (2006) The diverse functions of microRNAs in animal development and disease. Dev Cell 11: 441–450. 17011485 10.1016/j.devcel.2006.09.009

[9] Shin S, Cha HJ, Lee EM, Lee SJ, Seo SK, et al (2009) Alteration of miRNA profiles by ionizing radiation in A549 human non-small cell lung cancer cells. Int J Oncol 35: 81–86. 19513554

[10] Girardi C, De Pitta C, Casara S, Sales G, Lanfranchi G, et al (2012) Analysis of miRNA and mRNA expression profiles highlights alterations in ionizing radiation response of human lymphocytes under modeled microgravity. PLoS One 7: e31293. 22347458 10.1371/journal.pone.0031293 PMC3276573

[11] Niemoeller OM, Niyazi M, Corradini S, Zehentmayr F, Li M, et al (2011) MicroRNA expression profiles in human cancer cells after ionizing radiation. Radiat Oncol 6: 29. 21453501 10.1186/1748-717X-6-29 PMC3079656

[12] Cha HJ, Shin S, Yoo H, Lee EM, Bae S, et al (2009) Identification of ionizing radiation-responsive microRNAs in the IM9 human B lymphoblastic cell line. Int J Oncol 34: 1661–1668. 19424585 10.3892/ijo_00000297

[13] Jeong SH, Wu HG, Park WY (2009) LIN28B confers radio-resistance through the posttranscriptional control of KRAS. Exp Mol Med 41: 912–918. 19745602 10.3858/emm.2009.41.12.097 PMC2802686

[14] Oh JS, Kim JJ, Byun JY, Kim IA (2010) Lin28-let7 modulates radiosensitivity of human cancer cells with activation of K-Ras. Int J Radiat Oncol Biol Phys 76: 5–8. 20005451 10.1016/j.ijrobp.2009.08.028

[15] Arora H, Qureshi R, Jin S, Park AK, Park WY (2011) miR-9 and let-7g enhance the sensitivity to ionizing radiation by suppression of NFkappaB1. Exp Mol Med 43: 298–304. 21464588 10.3858/emm.2011.43.5.031 PMC3104252

[16] Lee KM, Choi EJ, Kim IA (2011) microRNA-7 increases radiosensitivity of human cancer cells with activated EGFR-associated signaling. Radiother Oncol 101: 171–176. 21676478 10.1016/j.radonc.2011.05.050

[17] Chen S, Wang H, Ng WL, Curran WJ, Wang Y (2011) Radiosensitizing effects of ectopic miR-101 on non-small-cell lung cancer cells depend on the endogenous miR-101 level. Int J Radiat Oncol Biol Phys 81: 1524–1529. 22014955 10.1016/j.ijrobp.2011.05.031

[18] Babar IA, Czochor J, Steinmetz A, Weidhaas JB, Glazer PM, et al (2011) Inhibition of hypoxia-induced miR-155 radiosensitizes hypoxic lung cancer cells. Cancer Biol Ther 12: 908–914. 22027557 10.4161/cbt.12.10.17681 PMC3280906

[19] Chu YW, Yang PC, Yang SC, Shyu YC, Hendrix MJ, et al (1997) Selection of invasive and metastatic subpopulations from a human lung adenocarcinoma cell line. Am J Respir Cell Mol Biol 17: 353–360. 9308922 10.1165/ajrcmb.17.3.2837

[20] Chang YW, Chen SC, Cheng EC, Ko YP, Lin YC, et al (2005) CD13 (aminopeptidase N) can associate with tumor-associated antigen L6 and enhance the motility of human lung cancer cells. Int J Cancer 116: 243–252. 15812828 10.1002/ijc.21089

[21] Gao M, Yeh PY, Lu YS, Chang WC, Kuo ML, et al (2008) NF-kappaB p50 promotes tumor cell invasion through negative regulation of invasion suppressor gene CRMP-1 in human lung adenocarcinoma cells. Biochem Biophys Res Commun 376: 283–287. 18782567 10.1016/j.bbrc.2008.08.144

[22] Liu YC, Yen HY, Chen CY, Chen CH, Cheng PF, et al (2011) Sialylation and fucosylation of epidermal growth factor receptor suppress its dimerization and activation in lung cancer cells. Proc Natl Acad Sci U S A 108: 11332–11337. 21709263 10.1073/pnas.1107385108 PMC3136320

[23] Tian T, Hao J, Xu A, Luo C, Liu C, et al (2007) Determination of metastasis-associated proteins in non-small cell lung cancer by comparative proteomic analysis. Cancer Sci 98: 1265–1274. 17537172 10.1111/j.1349-7006.2007.00514.x PMC11158557

[24] Lize M, Klimke A, Dobbelstein M (2011) MicroRNA-449 in cell fate determination. Cell Cycle 10: 2874–2882. 21857159 10.4161/cc.10.17.17181

[25] Lize M, Pilarski S, Dobbelstein M (2010) E2F1-inducible microRNA 449a/b suppresses cell proliferation and promotes apoptosis. Cell Death Differ 17: 452–458. 19960022 10.1038/cdd.2009.188

[26] Yang X, Feng M, Jiang X, Wu Z, Li Z, et al (2009) miR-449a and miR-449b are direct transcriptional targets of E2F1 and negatively regulate pRb-E2F1 activity through a feedback loop by targeting CDK6 and CDC25A. Genes Dev 23: 2388–2393. 19833767 10.1101/gad.1819009 PMC2764491

[27] Noonan EJ, Place RF, Pookot D, Basak S, Whitson JM, et al (2009) miR-449a targets HDAC-1 and induces growth arrest in prostate cancer. Oncogene 28: 1714–1724. 19252524 10.1038/onc.2009.19

[28] Bou Kheir T, Futoma-Kazmierczak E, Jacobsen A, Krogh A, Bardram L, et al (2011) miR-449 inhibits cell proliferation and is down-regulated in gastric cancer. Mol Cancer 10: 29. 21418558 10.1186/1476-4598-10-29 PMC3070685

[29] Lize M, Herr C, Klimke A, Bals R, Dobbelstein M (2010) MicroRNA-449a levels increase by several orders of magnitude during mucociliary differentiation of airway epithelia. Cell Cycle 9: 4579–4583. 21088493 10.4161/cc.9.22.13870

[30] Jeon HS, Lee SY, Lee EJ, Yun SC, Cha EJ, et al (2012) Combining microRNA-449a/b with a HDAC inhibitor has a synergistic effect on growth arrest in lung cancer. Lung Cancer 76: 171–176. 22078727 10.1016/j.lungcan.2011.10.012

[31] Marcet B, Chevalier B, Luxardi G, Coraux C, Zaragosi LE, et al (2011) Control of vertebrate multiciliogenesis by miR-449 through direct repression of the Delta/Notch pathway. Nat Cell Biol 13: 693–699. 21602795 10.1038/ncb2241

[32] Kozomara A, Griffiths-Jones S (2011) miRBase: integrating microRNA annotation and deep-sequencing data. Nucleic Acids Res 39: D152–157. 21037258 10.1093/nar/gkq1027 PMC3013655

[33] Lu TP, Lee CY, Tsai MH, Chiu YC, Hsiao CK, et al (2012) miRSystem: an integrated system for characterizing enriched functions and pathways of microRNA targets. PLoS One 7: e42390. 22870325 10.1371/journal.pone.0042390 PMC3411648

[34] Noonan EJ, Place RF, Basak S, Pookot D, Li LC (2010) miR-449a causes Rb-dependent cell cycle arrest and senescence in prostate cancer cells. Oncotarget 1: 349–358. 20948989 10.18632/oncotarget.167 PMC2952964

[35] Chang CC, Shih JY, Jeng YM, Su JL, Lin BZ, et al (2004) Connective tissue growth factor and its role in lung adenocarcinoma invasion and metastasis. J Natl Cancer Inst 96: 364–375. 14996858 10.1093/jnci/djh059

[36] Shih JY, Yang SC, Hong TM, Yuan A, Chen JJ, et al (2001) Collapsin response mediator protein-1 and the invasion and metastasis of cancer cells. J Natl Cancer Inst 93: 1392–1400. 11562390 10.1093/jnci/93.18.1392

[37] Ho CC, Huang PH, Huang HY, Chen YH, Yang PC, et al (2002) Up-regulated caveolin-1 accentuates the metastasis capability of lung adenocarcinoma by inducing filopodia formation. Am J Pathol 161: 1647–1656. 12414512 10.1016/S0002-9440(10)64442-2 PMC1850800

[38] Rofstad EK (1992) Radiation sensitivity in vitro of primary tumors and metastatic lesions of malignant melanoma. Cancer Res 52: 4453–4457. 1643637

[39] Suit H, Allam A, Allalunis-Turner J, Brock W, Girinsky T, et al (1994) Is tumor cell radiation resistance correlated with metastatic ability? Cancer Res 54: 1736–1741. 8137288

[40] Salem SD, Abou-Tarboush FM, Saeed NM, Al-Qadasi WD, Farah MA, et al (2012) Involvement of p53 in gemcitabine mediated cytotoxicity and radiosensitivity in breast cancer cell lines. Gene 498: 300–307. 22353361 10.1016/j.gene.2012.01.099

[41] Thomas CP, Buronfosse A, Portoukalian J, Fertil B (1995) Correlation between the radiosensitivity in vitro of clones and variants derived from a human melanoma cell line and their spontaneous metastatic potential in vivo. Cancer Lett 88: 221–225. 7874696 10.1016/0304-3835(94)03624-r

[42] Chaudhry MA, Sachdeva H, Omaruddin RA (2010) Radiation-induced micro-RNA modulation in glioblastoma cells differing in DNA-repair pathways. DNA Cell Biol 29: 553–561. 20380575 10.1089/dna.2009.0978

[43] Josson S, Sung SY, Lao K, Chung LW, Johnstone PA (2008) Radiation modulation of microRNA in prostate cancer cell lines. Prostate 68: 1599–1606. 18668526 10.1002/pros.20827 PMC3182144

[44] Yan D, Ng WL, Zhang X, Wang P, Zhang Z, et al (2010) Targeting DNA-PKcs and ATM with miR-101 sensitizes tumors to radiation. PLoS One 5: e11397. 20617180 10.1371/journal.pone.0011397 PMC2895662

[45] Balca-Silva J, Sousa Neves S, Goncalves AC, Abrantes AM, Casalta-Lopes J, et al (2012) Effect of miR-34b overexpression on the radiosensitivity of non-small cell lung cancer cell lines. Anticancer Res 32: 1603–1609. 22593438

[46] Wu Y, Liu GL, Liu SH, Wang CX, Xu YL, et al (2012) MicroRNA-148b enhances the radiosensitivity of non-Hodgkin's Lymphoma cells by promoting radiation-induced apoptosis. J Radiat Res 53: 516–525. 22843616 10.1093/jrr/rrs002 PMC3393342

[47] Hunakova L, Chorvath M, Duraj J, Bartosova Z, Sevcikova L, et al (2000) Radiation-induced apoptosis and cell cycle alterations in human carcinoma cell lines with different radiosensitivities. Neoplasma 47: 25–31. 10870683

[48] Quiet CA, Weichselbaum RR, Grdina DJ (1991) Variation in radiation sensitivity during the cell cycle of two human squamous cell carcinomas. Int J Radiat Oncol Biol Phys 20: 733–738. 2004949 10.1016/0360-3016(91)90016-w

[49] Tell R, Heiden T, Granath F, Borg AL, Skog S, et al (1998) Comparison between radiation-induced cell cycle delay in lymphocytes and radiotherapy response in head and neck cancer. Br J Cancer 77: 643–649. 9484824 10.1038/bjc.1998.103 PMC2149925

[50] Eriksson D, Stigbrand T (2010) Radiation-induced cell death mechanisms. Tumour Biol 31: 363–372. 20490962 10.1007/s13277-010-0042-8

[51] Lima RT, Busacca S, Almeida GM, Gaudino G, Fennell DA, et al (2011) MicroRNA regulation of core apoptosis pathways in cancer. Eur J Cancer 47: 163–174. 21145728 10.1016/j.ejca.2010.11.005

[52] Han Y, Zhang Y, Yang LH, Mi XY, Dai SD, et al (2012) X-radiation inhibits histone deacetylase 1 and 2, upregulates Axin expression and induces apoptosis in non-small cell lung cancer. Radiat Oncol 7: 183. 23110995 10.1186/1748-717X-7-183 PMC3542190

[53] Zhang B, Wang Y, Pang X (2012) Enhanced radiosensitivity of EC109 cells by inhibition of HDAC1 expression. Med Oncol 29: 340–348. 20464640 10.1007/s12032-010-9559-3

[54] Jeggo P, Lavin MF (2009) Cellular radiosensitivity: how much better do we understand it? Int J Radiat Biol 85: 1061–1081. 19995233 10.3109/09553000903261263

[55] Branzei D, Foiani M (2008) Regulation of DNA repair throughout the cell cycle. Nat Rev Mol Cell Biol 9: 297–308. 18285803 10.1038/nrm2351

