# Supplementary figures and images for: MicroRNA-449a Enhances Radiosensitivity in CL1-0 Lung Adenocarcinoma Cells
Source: PLoS One. 2013 Apr 17;8(4):e62383. doi: 10.1371/journal.pone.0062383 (PMC3629161; doi:10.1371/journal.pone.0062383)

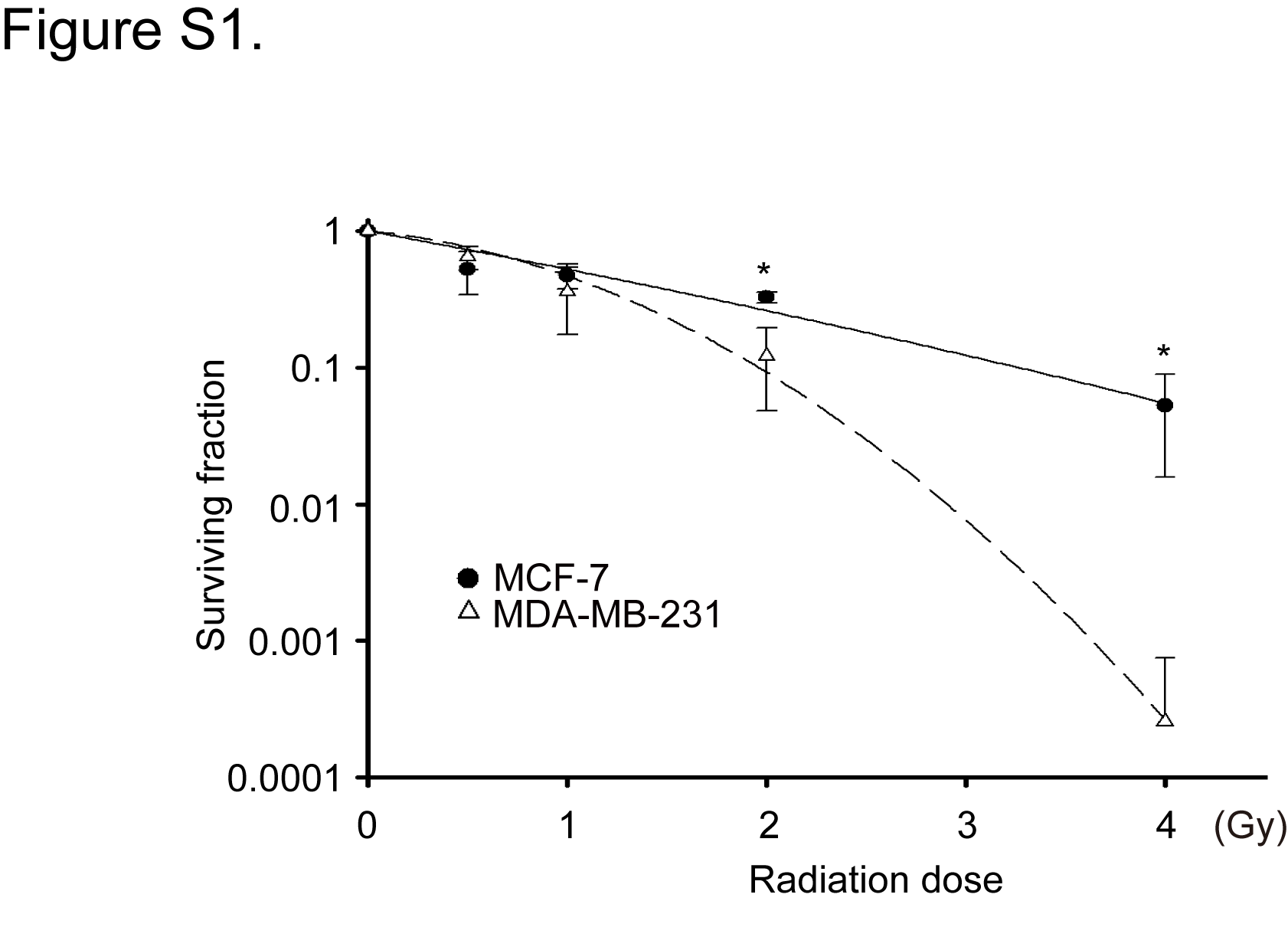

Supplement: Figure S1 — Differential irradiation responses in MCF7 and MDA-MB-231. Clonogenic assays of MCF7 and MDA-MB-231 cells treated with 0, 0.5, 1, 2, and 4 Gy radiation. The surviving fraction was measured 8 days post-irradiation. (TIF) [file pone.0062383.s001.tif]
